# Fingertips Ischemia, Nephroangiosclerosis, and Focal Segmental Glomerulosclerosis: Is Genetic Thrombophilia the Unique Explanation?

**DOI:** 10.1155/2014/832592

**Published:** 2014-03-17

**Authors:** Lisa Giovannini, Carlo Donadio

**Affiliations:** Department of Clinical & Experimental Medicine, School of Nephrology, University of Pisa, Via Paradisa 2, 56100 Pisa, Italy

## Abstract

*Case Presentation*. 53-years-old-man with essential hypertension and nonnephrotic proteinuria (1.3 gr/24 h) and with normal renal function (eGFR-MDRD 123 mL/min/1.73 m^2^) was admitted to nephrology department; kidney biopsy showed FSGS; two years later the patient presented with ulceration and ischemic gangrene of the IV and V right-hand fingertips; genetic analysis demonstrated polymorphism of the methylenetetrahydrofolate reductase genes C677T (heterozygote C677T/1298AC with normal value of homocysteine) and mutations of prothrombin gene G20210A and of plasminogen activator inhibitor-1 4G/5G 675 with slight increase of its value. After five years from biopsy, 24-hours proteinuria was still around 1–1.3 g/die; renal function was still normal (eGFR 107 ml/min/1.73 m^2^). These data are against the previous diagnosis of primary FSGS. We hypothesize that genetic thrombophilia may explain all the clinical signs of our patient. *Conclusions*. Alterations in genes of thrombophilia should be ruled out in patients with bioptic diagnosis of “primary” FSGS, in particular if clinically atypical.

## 1. Background

Focal segmental glomerulosclerosis is a histopathological pattern of lesions, where “focal” refers to involving minority of glomeruli and the “segmental” refers to involving a portion of the glomerular capillary tuft, caused by injury of podocytes. Clinically it manifests proteinuria which can progress to nephrotic syndrome and eventually to end stage renal failure. Some clinical and analytical data can be extremely helpful to differentiate primary or genetic forms of FSGS from hyperfiltering types.

A slow increase of proteinuria and the absence of hypoalbuminemia and edema even in the presence of massive proteinuria are very characteristic of hyperfiltering FSGS. This is in contrast with the rapid appearance of nephrotic syndrome and a rapid decrease of renal function in primary types.

The distinction between the different types of FSGS is crucial, since their treatment is radically different.

Thrombotic microangiopathy is a rare cause of adaptative FSGS due to inherited thrombophilia. The aim of this paper is to suggest that inherited thrombophilia should be considered as possible cause of secondary FSGS.

## 2. Case Presentation

In this paper we report the case of a 53-year-old Italian man (body weight 85 Kg, height 1.85 m, and BMI 24.8 kg/m^2^) with familiarity for cardiovascular and renal disease (the father, affected by hypertension and chronic kidney disease, died at 55 years due to acute myocardial infarction). The patient had been diagnosed as having moderate essential hypertension at the age of 42 and treated with combination therapy of calcium channel blockers (amlodipine 5 mg) and ACEI (ramipril 5 mg). At the age of 47 laboratory analyses demonstrated hypercholesterolemia (total cholesterol 270 mg/dL) and nonnephrotic proteinuria (1.3 gr/24 h) with normal renal function (serum creatinine 0.8 mg/dL, eGFR-MDRD 123 mL/min/1.73 m^2^).

**Figure 1:**
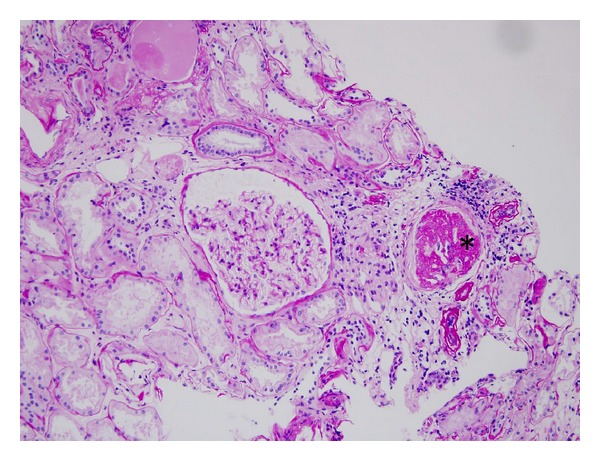
The glomerulus indicated by (*) is solid and hypercellular and surrounded by severe interstitial inflammatory infiltrate (20 × Periodic acid Schiff), 359 × 270 mm.

**Figure 2:**
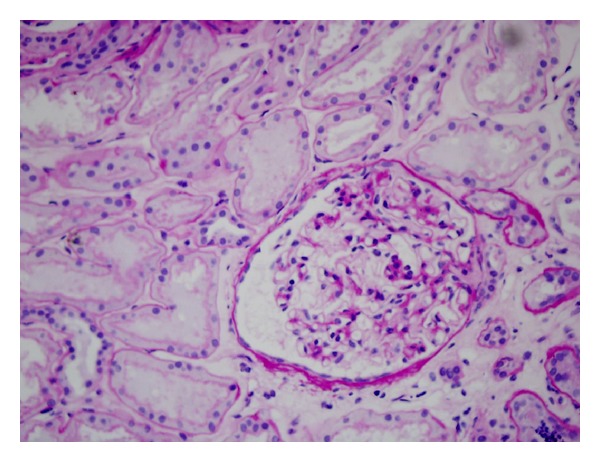
Most of glomeruli as this demonstrate only minor abnormalities (40 × Periodic acid Schiff), 359 × 270 mm.

The patient was admitted to a nephrology department where renal biopsy was performed. The biopsy material contained 19 glomeruli: global glomerular sclerosis and infiltration of lymphocytes were demonstrated in four glomeruli (Figure 1), small areas of segmental sclerosis were found in two other glomeruli, and the remaining 13 glomeruli showed only minor abnormalities (Figure 2). Rare signs of tubular atrophy, interstitial fibrosis, and interlobular sclerosis were also noted. Immunofluorescence examination did not show deposition of IgA, IgG, IgM, C1q, C3, k, and lambda fragments. The final diagnosis was primary focal and segmental glomerulosclerosis (FSGS). According to this diagnosis, the patient was treated with dietary sodium restriction, antihypertensive drugs (ramipril 5 mg and losartan 100 mg), and high dose glucocorticoid therapy (methylprednisolone 1000 mg ev once monthly for three months) followed by prednisone 1 mg/kg/die (75 mg), with progressive reduction of the dose, for 6 months. At the end of treatment, proteinuria was <0.5 g/die.

At the age of 49 the patient showed skin ulceration and ischemic gangrene of the IV and V right- hand fingertips. Serological tests for connective tissue diseases (*rheumatoid factor *RF,* antinuclear antibodies* ANA,* anticentromere antibodies* ACA,* Sclero-70* Scl-70,* antimitochondrial antibodies* AMA,* anti-smooth-muscle antibodies* ASMA, IgM and IgG* anticardiolipin antibodies* aCL, and* anti-*β**_2_ *-glycoprotein 1 antibodies *β**_2_GPI) and vasculitis (*antineutrophil cytoplasmic antibodies* ANCA c/p,* antiphospholipid antibodies* aPL, C1q antibodies, and* anti-phosphatidylserine-prothrombin antibodies* aPS-PT) were performed and excluded the major acquired thrombophilic disorders. Doppler ultrasound examination ruled out aneurysms or stenosis of the arteries of the right upper limb. Nailfold capillaroscopy showed aspecific alterations of the small vessel. The patient was treated with IV infusion of prostaglandins (prostacyclin 100 mcg in 50 mL of dextrose 5% in water/daily for two weeks), nitroglycerin patches on the skin of the two fingers (5 mg/24 h), and nadroparin calcium 5700 U.I./die for 4 months, with complete resolution of the gangrene. Genetic analysis demonstrated polymorphism of the genes MTHFR C677T (heterozygote C677T/1298AC with normal value of homocysteine) and mutations of prothrombin gene G20210A and of PAI-1 4G/5G 675 with slight increase of PAI-1 values. Screening for inherited thrombophilia demonstrated similar mutations in his 20-year-old daughter: homozygous 677 TT MTHFR with normal homocysteine (in treatment with folic acid) and slight increase of PAI-1 value.

On the basis of the genetic analyses the patient was treated with folic acid 400 mcg/die and pentoxifylline 800 mg for 6 months and had long-term treatment with acetylsalicylic acid 100 mg/die. The 53-year-old patient was evaluated in our nephrology clinic this year. Actually, arterial blood pressure is 140/90 mmHg in treatment with ramipril 5 mg and losartan 100 mg; urinalysis shows pH 5.5, urinary gravity 1020, proteins 30 mg/dL; 24-hour urinary proteins excretion is in the range of 1–1.3 g/die; renal function is still normal (sCr 0.76 mg/dL and eGFR 107 mL/min/1.73 m^2^). These data are against the previous diagnosis of primary FSGS. The aim of this paper is to suggest inherited thrombophilia as possible cause of secondary FSGS in this patient.

## 3. Discussion

Prothrombin (PT) (G20210A) [2], methylenetetrahydrofolate reductase (MTHFR) (C677T) [1], and Factor V Leiden (G1691A) [3] are well-recognized genetic risk factors for venous thrombosis, while their role in patients with arterial thrombosis remains to be clarified. Factor V Leiden, PT G20210A, and MTHFR C677T polymorphisms increase the risk for myocardial infarction, ischemic stroke, and peripheral vascular disease, particularly among younger patients and women [4]. It has also been demonstrated that G20210A PT allele represents an inherited risk factor for acute coronary syndrome [5]. Moreover patients with essential hypertension, homozygous C677TT, or doubly heterozygous C677CT/1298AC genotypes are predisposed to develop hypertensive nephrosclerosis and chronic renal failure [6]. Nephrosclerosis, which is common in chronic glomerulonephritis, may be determined by intraglomerular blood clotting, like in thrombotic microangiopathy [7].

Focal segmental glomerulosclerosis, once considered a single disease, is now viewed as a group of clinical-pathologic syndromes sharing a common glomerular lesion, mediated by diverse insults directed to the podocytes. Commonly, FSGS is classified as a primary, idiopathic form, mediated by circulating permeability factors, or as secondary form. Focal segmental glomerulosclerosis usually results in several settings mediated by glomerular hypertrophy hyperfiltration (e.g., oligonephronia, unilateral renal agenesis, or scarring due to reflux nephropathy or to systemic disease, such as severe obesity, hypertension, diabetes mellitus, vasculitis, lupus, and glomerular thrombotic microangiopathy in antiphospholipid syndrome [8]). Other known causes include HIV infection, heroin nephropathy, interferon, and pamidronato medications.

Primary FSGS is characterized by heavy proteinuria and progression of renal functional impairment, while secondary forms, in particular the adaptative form, show only modest proteinuria, slight progression of renal impairment, and a better prognosis [9].

It is important to distinguish primary from secondary FSGS because the treatment is radically different. Secondary FSGS's therapy consists of angiotensin inhibition than immunosuppressive therapy in the idiopathic form.

Our patient received a bioptic diagnosis of primary FSGS which appears atypical in both clinical and histologic features. Clinically, the patient is a normotype man (BMI 24.8 kg/m^2^), without urinary tract infections in the past; the ultrasound examination shows symmetric normal kidneys (12 cm–12.2 cm) with normal echogenicity; we excluded systemic lupus erythematosus, systemic sclerosis, antiphospholipid syndrome, and ANCA-associated small vessels vasculitis. The renal biopsy showed global sclerosis of 4 glomeruli and light focal alterations in only 2; moreover, the stability of the renal function, which remained normal during many years, and a moderate proteinuria ranging between 0.5 and 1.3 g/die suggest a secondary form of FSGS and a good prognosis. The patient has also many inherited and acquired cardiovascular risk factors, in particular some genetic abnormalities linked to thrombophilia. Based on these data and on the history of the ischemic injury of the fingertips of the right hand, possibly secondary to thrombosis of the small vessels, we hypothesize that the glomerulosclerosis may be secondary to two pathogenetic mechanisms: at first time glomerular microthrombotic events leading to glomerular sclerosis and finally scarring due to hypertensive nephroangiosclerosis. We suggest that both events (fingers gangrene and glomerulosclerosis) were determined by the genetic mutations of thrombophilic genes.

Indeed, the clinical data of our patient are in agreement with the recent report of association between chronic glomerulonephritis and inherited thrombophilia. Inherited thrombophilia occurred in eight of the 17 patients (47%) with idiopathic renal vascular disease: PT G20210A mutation, factor V Leiden mutation, and homozygosity for the MTHFR C677T polymorphism were found. Note that in 7/11 patients the bioptic diagnosis was FSGS. No patient had nephrotic syndrome and their renal function was moderately reduced (we calculated an average eGFR of 45 mL/min/1.73 m^2^, ranging between 15 and 79 mL/min/1.73 m^2^) [10, 11].

## 4. Conclusions

All together, the literature reports and the data of our patient suggest that referred inherited thrombophilic factors may lead to FSGS through a thrombotic microangiopathy and glomerular scarring due to hypertensive nephrosclerosis; moreover, this case report confirms that the reported mutation can lead to renal vascular sclerosis.

## List of Abbreviations

MDRD: Modified diet in renal disease
PT: Prothrombin
MTHFR: Methylenetetrahydrofolate reductase
FSGS: Focal segmental glomerulosclerosis.
